# A Potential Antitumor Effect of Dendritic Cells Fused with Cancer Stem Cells in Hepatocellular Carcinoma

**DOI:** 10.1155/2019/5680327

**Published:** 2019-04-01

**Authors:** Ye-Bin Pang, Jian He, Bi-Yu Cui, Sheng Xu, Xi-Lei Li, Man-Ya Wu, Rong Liang, Yan Feng, Xing Guo, Xue-Hui Zhang, Xiao-Ling Luo

**Affiliations:** ^1^Department of Gynecological Oncology, Affiliated Tumor Hospital of Guangxi Medical University, Nanning 530021, China; ^2^National Center for International Research of Biological Targeting Diagnosis and Therapy, Guangxi Medical University, Nanning 530021, China; ^3^Research Department, Affiliated Tumor Hospital of Guangxi Medical University, Nanning 530021, China; ^4^National Key Laboratory of Medical Immunology & Institute of Immunology, The Second Military Medical University, Shanghai, China; ^5^First Department of Chemotherapy, Affiliated Tumor Hospital of Guangxi Medical University, Nanning 530021, China

## Abstract

HCC stem cells were reported as posttreatment residual tumor cells that play a pivotal role in tumor relapse. Fusing dendritic cells (DCs) with tumor cells represents an ideal approach to effectively activate the antitumor immunity *in vivo*. DC/HCC stem cell vaccine provides a potential strategy to generate polyclonal immune response to multiple tumor stem cell antigens including those yet to be unidentified. To assess the potential capacity of DC/HCC stem cell vaccines against HCC, CD90^+^HepG2 cells were sorted from the HCC cell line HepG2. DC and CD90^+^HepG2 and DC and HepG2 fused cells were induced by polyethylene glycol (PEG). The influence of fusion cells on proliferation and immunological function transformation of lymphocytes was assessed by FCM and ELISA assay, respectively. The cytotoxicity assay of specific fusion cell-induced CTLs against HepG2 was conducted by CytoTox 96 Non-Radioactive Cytotoxicity Assay kit *in vitro*. At last, the prevention of HCC formation *in vivo* was described in a mouse model. The results of FCM analysis showed that the proportion of CD90^+^HepG2 cells in the spheral CD90^+^HepG2 enriched by suspension sphere culture was ranging from 98.7% to 99.5%, and 57.1% CD90^+^HepG2/DC fused cells were successfully constructed. The fusion cells expressed a higher level of costimulatory molecules CD80, CD83, CD86, and MHC-I and MHC-II molecules HLA-ABC and HLA-DR than did immature DCs (*P* < 0.05). And the functional analysis of fusion cell-induced CTLs also illustrated that CD90^+^HepG2/DC fusion cells showed a greater capacity to activate proliferation of lymphocytes *in vitro* (*P* < 0.05). The CD90^+^HepG2/DC-activated CTLs had a specific killing ability against CD90^+^HepG2 cells *in vivo*. These results suggested that CD90^+^HepG2/DC fusion cells could efficiently stimulate T lymphocytes to generate specific CTLs targeting CD90^+^HepG2 cells. It might be a promising strategy of immunotherapy for HCC.

## 1. Introduction

HCC is a common malignancy with a dismal prognosis. Only a minority of patients are eligible for surgery, and the five-year survival rate of HCC is less than 15% because of metastasis and recurrence [1, 2]. In addition, HCC cells are not sensible to chemotherapy and radiotherapy. Therefore, novel effective strategies are needed urgently for inhibiting the metastasis and recurrence of HCC.

In recent years, advances in understanding of HCC tumor biology [3–5] have proved that the metastasis and recurrence of HCC after surgery are closely related to cancer stem cells (CSCs). The CSCs are a small group of cells that are endowed with the ability to perpetuate themselves through self-renewal and to generate mature cells through differentiation, which are responsible for tumor formation and progression. These findings remind us that the surviving CSCs may be the cause of tumor relapse and the failure of cancer conventional therapy. Hence, targeting CSCs would be an effective therapeutic strategy to inhibit tumor relapse. According to previous studies [6–9], CD90^+^ phenotype cells have been considered CSCs in the HCC cell line because of the characteristics of greater colony-forming efficiency *in vitro*, higher proliferative ability, and greater tumorigenic capacity *in vivo* when compared to normal HCC cells.

Dendritic cells (DCs) are the most important potent antigen-presenting cells *in vivo*, which prominently express costimulatory molecules and are uniquely able to induce primary immune responses [10]. DC/tumor fusion cells, first reported by Gong et al. [11], can process and present a broad array of tumor antigens including tumor-associated Ag (TAA) and tumor-specific Ag (TSA) to autologous T cells and then induce effectively specific T cell immunity. Recently, it is reported by Li et al. [9] that DC vaccination using lung CSC antigens induced MHC expression, cytokine production, lymphocyte infiltration, and long-term protection against prostate cancer. Furthermore, it is likely that certain stem cell markers expressed by CSCs may possess distinct antigenicity and thus provide opportunities for enhanced immunotherapy. To date, the possibility of DCs loaded with CSC antigen in cancer immunotherapy has been proved. However, the efficiency of fusing DCs with CSCs is not in the literature yet. In this study, we developed a DC/CSC vaccine and assessed the CTL responses to HCC CSCs.

## 2. Materials and Methods

### 2.1. Tumor Cell Lines

The human HCC cell line HepG2 was obtained from the American Type Culture Collection (ATCC). HepG2 cells were cultured in RPMI-1640 medium (Gibco Invitrogen, Carlsbad, CA, USA) with 10% (*v*/*v*) fetal bovine serum and 100 U/ml penicillin/streptomycin at 37°C in a humidified atmosphere supplemented with 5% CO_2_.

### 2.2. CSC Sorting and Enrichment by Suspension Sphere Culture

Cell sorting was performed by flow cytometry on a BD FACSVantage SE system (BD Biosciences, San Diego, USA). The HepG2 cells were labeled with an FITC-conjugated anti-CD90 antibody (BD Biosciences, San Diego, USA) according to the manufacturer's instructions. Then, the purity of the sorted cells was estimated by flow cytometry. According to the CSC enrichment method of suspension sphere culture from previous studies [12], the sorted CD90^+^HepG2 cells were first washed with PBS to remove serum and then suspended in serum-free media with IGF, EGF (20 ng/ml) (R&D Systems, Minneapolis, USA), and B27 (20 ml/l) (Gibco Invitrogen, Carlsbad, CA, USA). The suspended cells were plated in ultralow attachment 25 cm^2^ culture bottles (Corning Inc., NY, USA) at a density of 1000 cells/ml. The tumor sphere formation was observed under an inverted light microscope at 400x magnification. The spheres were collected at 800 rpm centrifugation and then dissociated with Accutase® (Sigma-Aldrich Co., St. Louis, MO, USA). Then, the resulting single-cell suspension was centrifuged and resuspended in serum-free medium to continue the formation of spheres. The sphere passage was administrated every 5-8 days.

### 2.3. Generation of Dendritic Cells and T Cells

PBMCs were isolated from HLA-A2^+^ healthy donors by Ficoll-Hypaque (Sigma-Aldrich Co., St. Louis, MO, USA) density gradient centrifugation and were incubated in six-well polystyrene plates at 37°C in 5% CO_2_ for 2 h in RPMI-1640. After that, nonadherent cells were removed and purified by a nylon wool column and subsequently cultured in RPMI-1640 complete medium with 20 U/ml rhIL-2 (PeproTech Inc., Rocky Hill, USA) as a source of autologous T cells. Adherent cells were replenished with RPMI-1640 complete medium containing 100 ng/ml granulocyte-macrophage colony-stimulating factor (GM-CSF) (PeproTech Inc., Rocky Hill, USA) and 50 ng/ml rh-interleukin-4 (IL-4) (PeproTech Inc., Rocky Hill, USA). Half of the culture medium and cytokines were replaced every 2 days. Suspension and loosely adherent cells were harvested on day 7.

### 2.4. DC/Tumor Fusion Cell Preparation

DCs were stained with the green fluorescent dye CFSE (Sigma-Aldrich) and coincubated with CD90^+^HepG2 and HeG2 (irradiated at 5000 cGy) stained with the red fluorescent dye PKH26 (Sigma-Aldrich) at a DC : tumor ratio of 2 : 1, using 50% PEG (PEG1450, Sigma-Aldrich) to boost the fusion process as described elsewhere [13]. Briefly, the mixed cell suspension was washed with serum-free RPMI-1640 prewarmed at 37°C and centrifuged at 1000 rpm for 5 min. After that, PEG was added to resuspend the pellets for 1 min and the pellets were stirred gently for 2 min. Then, the PEG was washed with serum-free RPMI-1640 3 times, and the fusion cells were incubated in complete RPMI-1640 medium with GM-CSF, IL-4, and maturation cytokine tumor necrosis factor-*α* (TNF-*α*, PeproTech) at 37°C with 5% CO_2_. The fusion rate was evaluated by flow cytometry analysis such that hybrid cells displaying both green and red fluorescence were considered fusion cells. The cell surface molecules including CD80, CD83, CD86, and HLA-ABC and HLA-DR that underwent change in expression were also estimated by flow cytometry.

### 2.5. Analysis of IL-12p70 Release by ELISA

The level of IL-12p70 released by mature DCs (2 × 10^6^/well) in the supernatants was evaluated using the protocol with the Human IL-12p70 ELISA Kit (Neobioscience Technology, China). The optical density (OD) was read at 450 nm using a microtiter plate reader.

### 2.6. T Lymphocyte Proliferation Assay

T cell proliferation detection was carried out by flow cytometry analysis as described previously [14]. In brief, allogenic T cells were isolated from nonadherent PBMCs through nylon wool columns and resuspended in PBS at a final concentration of 5 × 10^6^ cells/ml. Next, cells were labeled with 0.5 *μ*M of PKH26 in PBS for 10 min. 1 × 10^6^ stained T cells were taken out as parent generation, and the rest T cells were seeded in round-bottomed 6-well plates and cocultured with CD90^+^HepG2/DC fusion cells, HeG2/DC fusion cells, DC+HepG2 mixed cells (mDCs), and DCs alone at a DC : T cell ratio of 30 : 1 for 5 days at 4°C in light-proof condition. After 5 days, T cells were analyzed on live gating based on forward scatter/side scatter.

### 2.7. IFN-*γ* Enzyme-Linked Immunosorbent Spot Assay

For estimating the specific T cell responses, a test for IFN-*γ* production was carried out in an ELISpot kit (R&D Systems, Minneapolis, USA) according to the previous studies [15, 16]. Briefly, serum-free RPMI-1640 was preadded to the 96-well ELISpot plate to activate the specific IFN-*γ* antibody coated on the flat bottom of the plate for 10 min. Then, the purified T cells were coincubated with CD90^+^HepG2/DC, HeG2/DC fusion cells, mDCs, and DCs alone at a DC : T cell ratio of 10 : 1 for 18 h at 37°C in 5% CO_2_. 18 h later, the wells were decanted and treated with 200 *μ*l/well of ice-cold deionized water for 15 min to lyse the residual cells and washed 5 times with PBS. The ELISpot plate was further incubated with a biotinylated detector antibody for 1 h. The plate was washed 5 times with PBS again and incubated with streptavidin for 1 h. Finally, the plate was washed 3 times and developed with 3-amino-9-ethylcarbazole (AEC) for 30 min, after which each well was examined for positive spots visually and with a KS ELISpot system with version 4.3 software (Carl Zeiss, Hallbergmoos, Germany).

### 2.8. Cytotoxicity Assay of Specific CTLs

The cytotoxicity assays were conducted using the CytoTox 96 Non-Radioactive Cytotoxicity Assay kit (Promega, Madison, WI, USA). According to the protocol, autologous lymphocytes were cocultured with stimulator cells (CD90^+^HepG2/DC, HeG2/DC fusion cells, mDCs, and DCs alone) in complete RPMI-1640 containing 20 U/ml IL-2 for 7 days to generate specific CTLs. Then, the CTLs were harvested and coincubated with target cells CD90^+^HepG2 cells and HeG2 cells in a 96-well U-bottom plate at various effector : target ratios of 10 : 1, 30 : 1, and 100 : 1 for 4 h. Subsequently, 50 *μ*l per well of supernatants was collected for detecting lactate dehydrogenase (LDH) release in the Microplate Imaging System at an absorbance of 490 nm. As controls, the spontaneous release of LDH was evaluated by incubation of CTLs or target cells alone, and the maximum release of LDH was assessed by incubating target cells in 0.1% Triton X-100. The results of specific LDH release were calculated as follows: percent  specific  release = (experimental OD − effector  spontaneous  OD − target  spontaneous  OD)/(target  maximum  OD − target  spontaneous  OD).

### 2.9. *In Vivo* Studies

Female BALB/c nude mice, aged 4 to 6 weeks, were obtained from the Medical Experimental Animal Center of Guangxi Medical University and maintained under standard conditions described by the Guangxi Laboratory Animal Center. Mice were randomly divided into five groups (6 mice each), and the five groups were divided as follows: 1 × 10^6^ HepG2 mixed with 3 × 10^7^ CD90^+^HepG2-DC-CTLs, 3 × 10^7^ HepG2-DC-CTLs, 3 × 10^7^ mDC-CTLs, and 3 × 10^7^ DC-CTLs in a 100 *μ*l volume as (1) the CD90^+^HepG2-DC-CTL group, (2) HepG2-DC-CTL group, (3) mDC group, and (4) DC-CTL group, respectively, in a 100 *μ*l volume, and the suspended cells were injected subcutaneously into nude mice; also, 1 × 10^6^ HepG2 were suspended in 100 *μ*l PBS and injected subcutaneously into nude mice as (5) the control group. The tumor volumes were measured with a caliper once a week and evaluated as tumor volume (mm^3^) = length × width^2^/2. 12 weeks after cell injection, nude mice were sacrificed.

### 2.10. Statistical analysis

Data were presented as mean ± standard deviation. One-way ANOVA and Fisher's Least Significant Difference Test were carried out to estimate the difference within each group for DC surface molecules, T cell proliferation assays, IL12p70 and IFN-*γ* analysis, and cytotoxicity assays. Difference in the tumor volume among groups was evaluated by repeated-measures ANOVA and Fisher's Least Significant Difference for comparisons. All statistical analyses were performed in the SPSS 16.0 software package. Statistical significance was considered at *P* value < 0.05.

## 3. Result

### 3.1. CD90^+^HepG2 Cell Sorting and Enrichment

**Figure 1:**
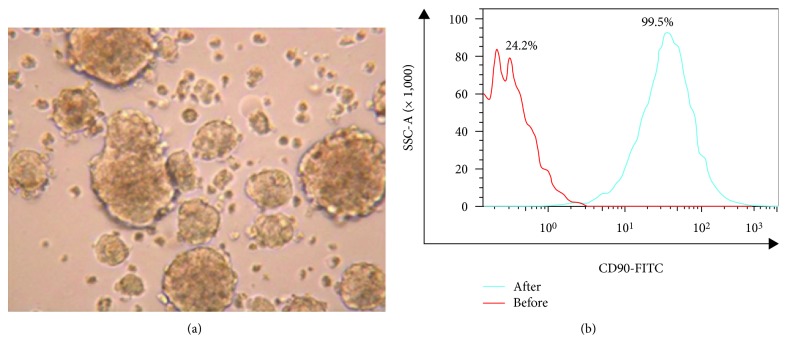
The HCC stem cells enriched by suspension sphere culture and the different expression of stem cell markers before and after enrichment. (a) CD90^+^HepG2 formed the anchorage-independent self-renewing spheres in the stem cell medium (×400). (b) The expression of CD90 on the HepG2 cell surface after enrichment was higher than that before enrichment by flow cytometry estimate. *P* < 0.001.

HepG2 were purified by flow cytometry, and 24.2% most brightly stained CD90^+^HepG2 cells were sorted. Then, the sorted CD90^+^HepG2 cells were enriched by suspension sphere culture [12]. The CD90^+^HepG2 cells generally formed nonadherent three-dimensional sphere clusters (Figure 1(a)). After that, the sphere clusters were harvested and then the proportion of CD90^+^HepG2 cells was detected. The average proportion was 99.5 ± 1.3% (Figure 1(b)).

### 3.2. Fusion Rate of DC/HepG2 and DC Phenotype

**Figure 2:**
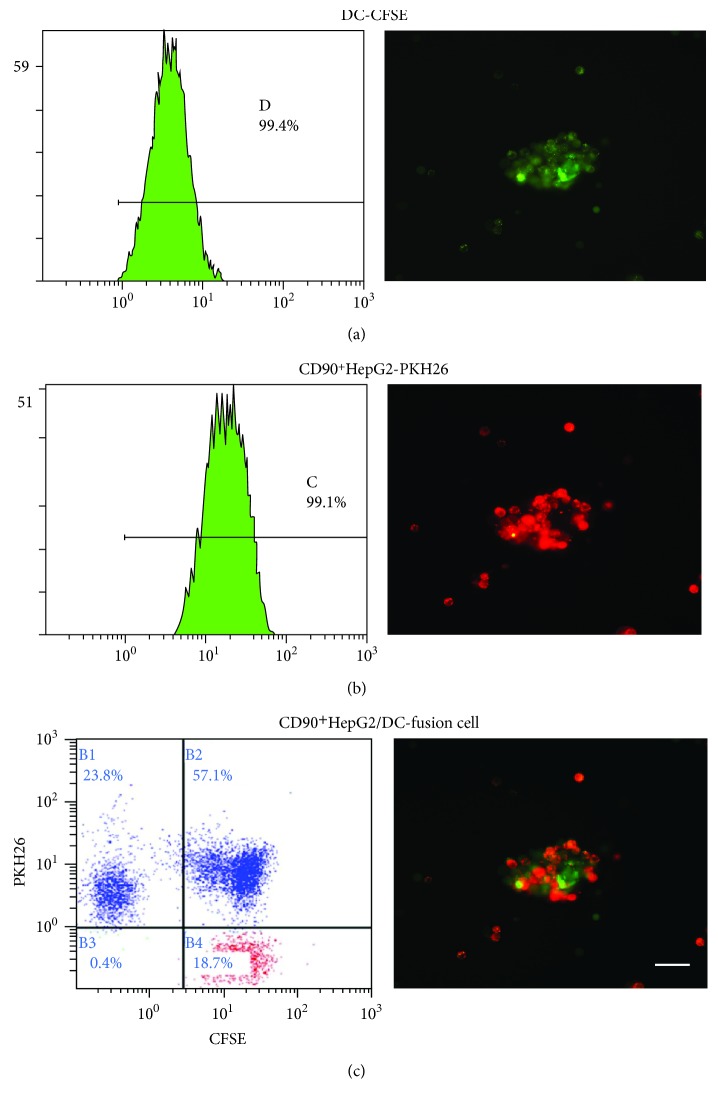
The immunofluorescent staining of DC and CD90^+^HepG2 and the fusion rate estimated by FCM. The fusion rate was estimated by immunofluorescence and FCM. (a) Dendritic cell (green), ×100, FCM analysis shows that the CFSE-positive rate is 99.4%. (b) CD90^+^HepG2 (red), ×100, FCM analysis shows that the PKH26-positive rate is 99.1%. (c) Fusion cells (green and red), ×100, FCM analysis shows that the double-positive rate is 57.1%.

**Figure 3:**
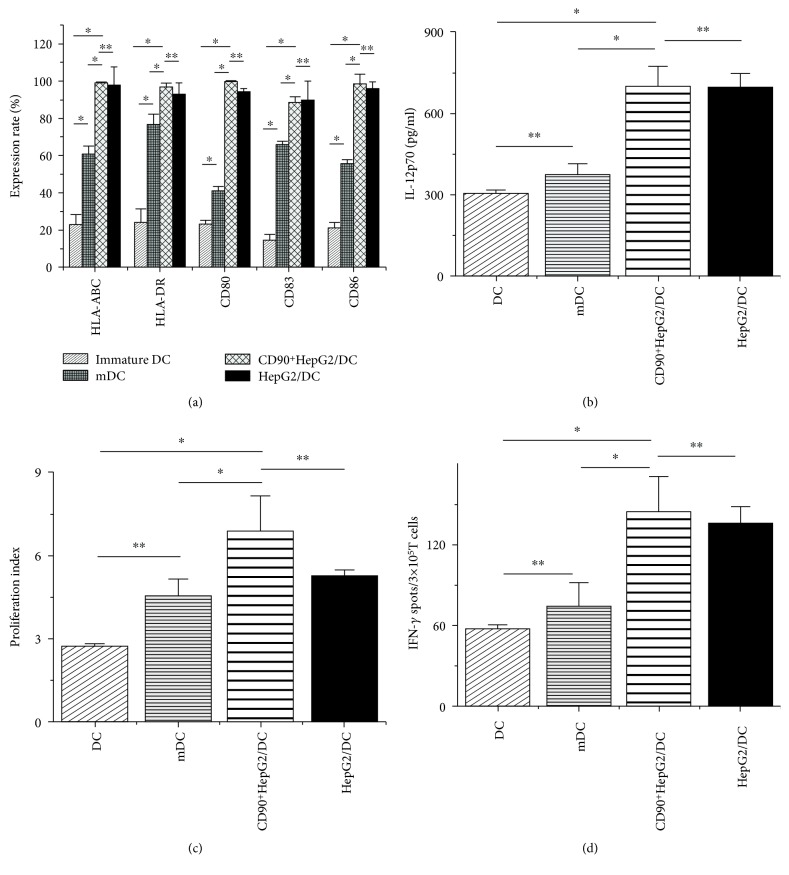
Flow cytometry analysis of expression change of surface molecules on immature DCs, mDCs, and fusion DCs and the immunological function of fusion cells. (a) The expression change of the costimulatory molecule and mature molecule on the cell surface of different DCs. ^∗^*P* < 0.01, ^∗∗^*P* > 0.05, ^∗∗∗^*P* value (the same as the first group). (b) The IL-12p70 level after 24 h culture in the supernatants from the four groups. ^∗^*P* < 0.05, ^∗∗^*P* > 0.05. (c) The different proliferation indices of the DCDC group, mDC group, HepG2/DC group, and CD90^+^HepG2/DC group. ^∗^*P* < 0.05, ^∗∗^*P* < 0.01, ^∗∗∗^*P* > 0.05. (d) IFN-*γ* ELISpot analysis of T cell IFN-*γ* secretion induced by the four groups of DCs. ^∗^*P* > 0.05, ^∗∗^*P* < 0.05.

The CFSE-positive DC was displayed as green (Figure 2(a)), and the PKH26-positive CD90^+^HepG2 was red (Figure 2(b)). The result showed that the fusion rate of CD90^+^HepG2/DC was 57.1% (Figure 2(c)) and the fusion rate of HepG2/DC was 55.4% (data not shown). Phenotypic changes in the expression of cell surface costimulatory molecules of fused DCs were also analyzed by flow cytometry. The expression of costimulatory molecules CD80, CD83, CD86, and major histocompatibility (MHC) molecules HLA-ABC and HLA-DR on fusion DCs was higher than that on mDCs and DCs alone (*P* < 0.05) (Figure 3(a)). Otherwise, there was no significant difference between CD90^+^HepG2/DC and HepG2/DC.

### 3.3. IL-12p70 Production

The IL-12p70 level in the supernatants of the four groups of DCs was evaluated. The IL-12p70 production of CD90^+^HepG2/DCs, HepG2/DCs, mDCs, and DCs alone was 701.15 ± 73.1 pg/ml, 698.47 ± 48.76 pg/ml, 375.52 ± 40.5 pg/ml, and 306.64 ± 10.4 pg/ml, respectively. The difference between fusion cells and mDC and fusion cells and DC was significant (*P* < 0.05) (Figure 3(b)). Otherwise, the difference between the two groups' fusion cells was not significant.

### 3.4. Analysis of Proliferation and IFN-*γ* Release of Allogeneic T Cells

The flow cytometry analysis of T cell proliferation showed that the proliferation index (PI) of T cells in the CD90^+^HepG2/DC group, HepG2/DC group, mDC group, and DC group was 6.92 ± 1.28, 5.31 ± 0.21, 4.6 ± 0.57, and 2.75 ± 0.073, respectively. The results demonstrated that the capacities of fusion cells to stimulate T cell proliferation were stronger than those of mDCs and DCs (Figure 3(c)). Furthermore, the result of IFN-*γ* release showed that the capacity of cytokine secretion of fusion cell-induced T cells was significantly stronger than that of the others (Figure 3(d)). The PI and IFN-*γ* release level of the CD90^+^HepG2/DC group were higher than those of the HepG2/DC group, but the difference was still not significant.

### 3.5. CD90^+^HepG2/DC-CTLs Have More Cytotoxicity Effect on CD90^+^HepG2 Cells

**Figure 4:**
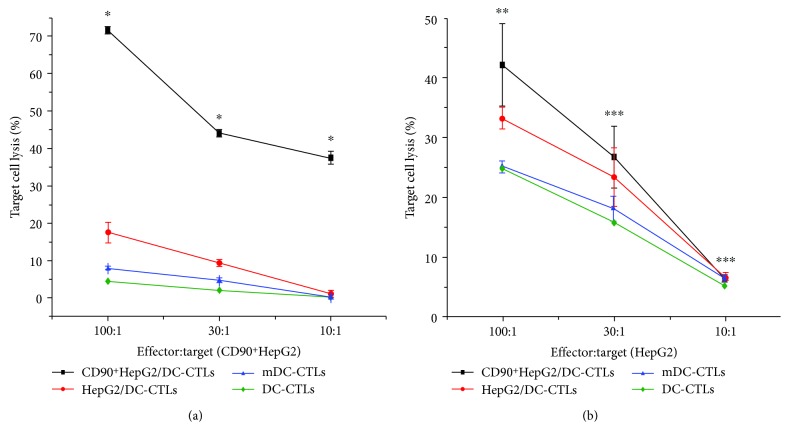
CD90^+^HepG2/DC-CTLs have more cytotoxicity effect on CD90^+^HepG2 cells. (a) Lytic activity specific to various CTLs against CD90^+^HepG2 cells. (b) Lytic activity specific to various CTLs against HepG2 cells. ^∗^*P* < 0.001, ^∗∗^*P* < 0.05, ^∗∗∗^*P* > 0.05.

The cytolysis capacities of CD90^+^HepG2/DC-CTLs, HepG2/DC-CTLs, mDC-CTLs, and DC-CTLs targeting CD90^+^HepG2 cells and HepG2 cells were evaluated. The result showed that CD90^+^HepG2/DC-CTLs targeted CD90^+^HepG2 cells most efficiently among the other groups at all effector : target ratios including 10 : 1, 30 : 1, and 100 : 1 (*P* < 0.001) (Figure 4(a)). However, the effect of CD90^+^HepG2/DC-CTLs targeting HepG2 cells was not better than that of other groups at the effector : target ratios of 10 : 1 and 30 : 1 (*P* > 0.05), except that the effector : target ratios went to 100 : 1 (Figure 4(b)) (Table 1).

### 3.6. Antitumor Immunity *In Vivo* Induced by CD90^+^HepG2/DC-CTLs

**Figure 5:**
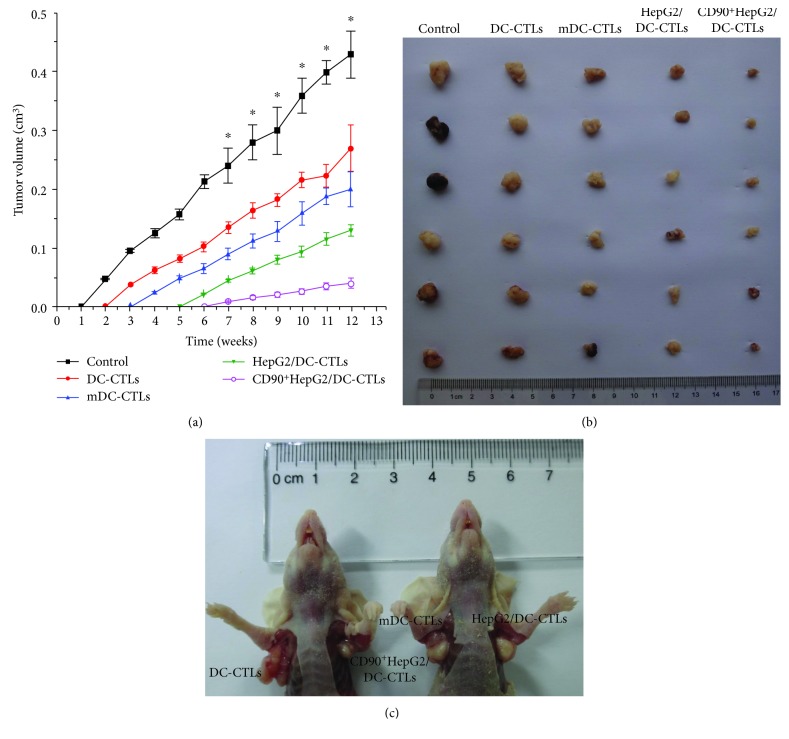
HepG2 cells mixed with CD90^+^HepG2/DC-CTLs, HepG2/DC-CTLs, mDC-CTLs, DC-CTLs, or PBS were injected into nude mice. (a) The time of tumor nodule becoming detectable in the CD90^+^HepG2/DC-CTLs group was significantly delayed. (b) The various tumor sizes of different groups and representative examples of nude mice. (c) CD90^+^HepG2/DC-CTLs inhibited tumor growth most efficiently compared to the DC-CTL, mDC-CTL, and HepG2/DC-CTL groups after injection. ^∗^*P* < 0.05.

To investigate whether CD90^+^HepG2/DC-CTLs against CSCs may inhibit HepG2 cell-induced tumor growth in vivo, CD90^+^HepG2/DC-CTLs, HepG2/DC-CTLs, mDC-CTLs, and DC-CTLs were injected into nude mice subcutaneously mixed with HepG2 cells. Compared to the other groups, the time of tumor nodule becoming detectable in the CD90^+^HepG2/DC-CTLs group was significantly delayed (Figure 5(a)). The detectable tumor nodules formed within 1 week in the control group, but took 2 weeks, 3 weeks, 5 weeks, and 6 weeks to form among DC-CTL, mDC-CTL, HepG2/DC-CTL, and CD90^+^HepG2/DC-CTL groups after injection, respectively (Figure 5(a)). At the end of the in vivo experiment, the tumor sizes of the CD90^+^HepG2/DC-CTL group were the smallest among the four groups and the difference became significant at 7 weeks after injection (Figures 5(b) and 5(c)).

## 4. Discussion

Multiple mechanisms may be involved in the development and progression of HCC, including the existence of HCC stem cells and the defects in the immune responses to these cells. One strategy to correct such defects is to enhance the tumor antigen presentation with the use of DCs. There is ample evidence that fusion of DCs with tumor cells is an effective approach for introducing tumor antigens into DCs. The tumor/DC fusion cell expresses the whole set of tumor antigens in the setting of costimulatory and MHC molecules and facilitates access of tumor antigens to endogenous and exogenous pathways of antigen presentation, resulting in the induction of both CD4 and CD8 T cells. In the present study, CD90^+^HepG2 cells were fused with DCs as a CD90^+^HepG2/DC vaccine. CD90^+^HepG2/DC fusion cells were applied as a whole-cell approach allowing for the presentation of CD90^+^ CSC antigen and multiple antigens including those yet to be identified. The results of the *in vitro* cytotoxicity assay of this work demonstrated that the CD90^+^HepG2/DC fusion cell approach not only developed the ability to target HCC CSCs but also effectively targeted ordinary HepG2 cells. The results of flow cytometry showed that the fusion rate of vaccine was 57.1%, and the fusion cells had a higher expression level of DC mature molecules CD83 and HLA-DR and costimulatory molecules CD80 and CD86 than did immature DCs, which demonstrated that the strategy of the CD90^+^HepG2/DC vaccine was practicable.

The unique functional criteria for tumor/DC fusion cells used in the vaccine settings are their capacity to secrete cytokines stimulating autologous immunity and activate cytotoxic T lymphocytes. For instance, IL-12 is a heterodimeric cytokine that upregulates DC expression of costimulatory molecules, stimulates T helper-1 reactivity, expands antigen-specific CD8^+^ T cells [14], and enhances the effectiveness of DC-based antitumor vaccines [13, 17]. On the other hand, the present studies [15, 18, 19] demonstrated evidence of immunological response to fusion cell vaccines by an increased expression of intracellular IFN-*γ*. In our study, the level of IL-12p70 in the fusion cell groups was higher than that in the mDC and DC groups. Meanwhile, the ability of the fusion cell groups to stimulate T cells was stronger than that of the other two groups, and the levels of IFN-γ in fusion cell-CTL groups were higher than those in mDC-CTL and DC-CTL groups. However, there was no significant difference in the experimental results above between the two fusion cell groups. This phenomenon was indicative of the similarity in cytokine-secreting ability, costimulatory molecule expression, and capacity to stimulate T cells between these two fusion cell groups.

It was obvious that CD90^+^HepG2/DC-CTLs targeting CD90 + HepG2 cells have the most efficiency according to the results of the cytotoxicity assay, which indicated that the DCs obtained stem-like TAAs during the fusion process and then presented the antigens to T cells, activating T cells to be cytolysis-specific CTLs. Meanwhile, not only was the time to form detectable tumor nodules in CD90^+^HepG2/DC-CTL group mice delayed compared to other groups, but also the tumor sizes were the smallest among all groups. The most reasonable explanation is that the CD90^+^HepG2/DC vaccine specifically killed CD90^+^HepG2 stem-like cells, reducing the number of HCC-initiating cells, which delayed the tumor formation. Following the reduction of CD90^+^HepG2 cells, the renewal and proliferation of HCC cells were also ceasing, leading to the final tumors being small in size. On the other hand, these results proved the stemness characteristic of CD90^+^HepG2 cells. Interestingly, we unexpectedly found in the cytotoxicity assay that the CD90^+^HepG2/DC-CTLs target HepG2 cells more efficiently than HepG2/DC-CTLs at the effector : target ratio of 100 : 1, but not different at other ratios. We guessed that when the effectors reached a certain amount, the CD90^+^HepG2/DC vaccine could kill the few CD90^+^HepG2 cells in a group of HepG2 cells. Unfortunately, we had not yet revealed the detailed mechanism how CTLs kill CSCs in this study. Questions remain regarding mechanisms underlying the apparent superior outcomes from DC vaccination targeting CSC. Yang et al. [3, 13, 20] pointed out that the stemness of CD90^+^HCC might relate to the Wnt/*β*-catenin, Hedgehog/SMO, Oct3/4, and Notch pathways; however, how the CD90^+^HepG2/DC vaccine works in communicating with the pathways is still unknown. Further multicenter research studies are needed to reveal the unknown mechanism in the future.

## Figures and Tables

**Table 1:** Different killing rates of the four groups of CTLs targeting CD90^+^HepG2 and HepG2 cells at various effector/target ratios.

Effector : target	CD90^+^HepG2/DC-CTLs		HepG2/DC-CTLs		mDC-CTLs		DC-CTLs	
	HepG2	CD90^+^HepG2	HepG2	CD90^+^HepG2	HepG2	CD90^+^HepG2	HepG2	CD90^+^HepG2
100 : 1	42.09 ± 6.93%^b^	71.51 ± 1.00%^c^	33.08 ± 1.67%^b^	17.48 ± 2.76%^c^	25.16 ± 0.94%^b^	7.91 ± 0.31%^c^	24.76 ± 1.12%^b^	4.47 ± 0.14%^c^
30 : 1	26.73 ± 5.19%^a^	43.97 ± 1.04%^c^	23.41 ± 4.87%^a^	9.63 ± 0.86%^c^	18.12 ± 2.08%^a^	4.75 ± 0.67%^c^	15.89 ± 0.13%^a^	1.99 ± 0.66%^c^
10 : 1	6.32 ± 0.34%^a^	37.42 ± 1.59%^c^	6.84 ± 0.77%^a^	1.25 ± 0.47%^c^	6.47 ± 0.58%^a^	0.15 ± 0.05%^c^	5.27 ± 0.22%^a^	0.37 ± 0.08%^c^

^a^ *P* > 0.05, ^b^*P* < 0.05, ^c^ *P* < 0.001.

## Data Availability

The pictures and tables used to support the findings of this study have been deposited in the Scientific Data's list of recommended repositories (DOI: 10.6084/m9.figshare.6818276).
